# CD2AP is a potential prognostic biomarker of renal clear cell carcinoma

**DOI:** 10.1002/cam4.7055

**Published:** 2024-03-08

**Authors:** Can Chen, Jia Xu, Jie‐Xin Zhang, Lin‐Yuan Chen, Yu‐Ang Wei, Wei‐Ming Zhang, Peng‐Fei Shao, Hua‐Guo Xu

**Affiliations:** ^1^ Department of Laboratory Medicine the First Affiliated Hospital of Nanjing Medical University Nanjing China; ^2^ Branch of National Clinical Research Center for Laboratory Medicine Nanjing China; ^3^ Department of Urology the First Affiliated Hospital of Nanjing Medical University Nanjing China; ^4^ Department of Pathology the First Affiliated Hospital of Nanjing Medical University Nanjing China

**Keywords:** ccRCC, CD2AP, DNA methylation, risk model

## Abstract

**Background:**

CD2‐associated protein (CD2AP) is a podocyte‐associated gene and its reduced expression is associated with the development of proteinuria and glomerulosclerosis. However, few studies have focused on the correlation between the expression and prognosis of CD2AP in renal clear cell carcinoma (ccRCC). Therefore, we aimed to assess the regulation of CD2AP expression and prognostic value in ccRCC.

**Methods:**

Multiple databases were employed to examine the expression of CD2AP in ccRCC. RT‐qPCR, Western Blot and immunohistochemistry were used to validate CD2AP expression in different cell lines and tissue samples. Kaplan–Meier analysis and ROC curve analysis were performed on the predictive prognostic performance of CD2AP. COX regression was used to construct CD2AP‐related prognostic models. The TIMER and TISIDB databases were used to analyze the correlation of tumor‐infiltrating immune cells with gene expression, mutations, somatic copy number variation, and immune molecules. Mass spectrometry was used to detect methylation status of the promoter CpG site of CD2AP in multiple cells.

**Results:**

We found that CD2AP expression was downregulated in ccRCC and its lower expression level was correlation with worse patient prognosis, higher tumor stage and grade and distant metastasis through analysis of databases, ccRCC cell lines and clinical tissue samples. Moreover, database and mass spectrometry techniques identified and validated cg12968598 hypermethylation as one of the key reasons for the downregulation of CD2AP expression. CD2AP expression was also associated with macrophage and neutrophil infiltration.

**Conclusions:**

Taken together, our results suggest that CD2AP can be used as a diagnostic and prognostic biomarker in ccRCC patients and that DNA hypermethylation plays an important role in reducing CD2AP expression.

## 1 INTRODUCTION

Clear cell renal cell carcinoma (ccRCC) is the most common type of renal cell carcinoma pathology and is usually treated by local excision, chemotherapy or radiotherapy, but some ccRCC patients still have a poor prognosis with distant metastases or local recurrence of ccRCC.^1^, ^2^ In recent years, studies have proposed that molecular targeted therapy can be performed on ccRCC patients to improve the prognosis of patients.^3^, ^4^ Hence, elucidation of the molecular mechanisms underlying the development of ccRCC and screening for new molecular markers suitable for early diagnosis and treatment of ccRCC is warranted.

CD2‐associated protein (CD2AP) acts as a bridging protein for CD2 cytoplasmic structural domain interactions, enhancing CD2 aggregation and anchoring it at cell contact sites, thereby stabilizing the interaction between T cells and antigen‐presenting cells.^5^, ^6^ CD2AP is highly expressed in immune cells, epithelial cells and neurons.^6^ Loss of function or gene mutation of CD2AP expressed in neurons and microglia of the brain lead to a series of pathological changes in the brain including amyloidogenesis and Tau‐mediated neurotoxicity, and ultimately lead to the formation of Alzheimer's disease.^7^, ^8^ In the kidney, CD2AP is mainly expressed in podocytes, and is used as an adaptor molecule to bind with nephrin and podocin, which are podocyte‐related proteins.^9^ This is essential for maintaining the function of the podocyte slit diaphragm and the glomerular filtration membrane.^10^ When CD2AP expression is reduced or mutated, the podocytes are damaged and the glomerular filtration rate is reduced, leading to kidney diseases such as proteinuria, glomerulosclerosis and renal insufficiency.^7^, ^11^

DNA methylation is a common mechanism in the epigenetic regulation of genes.^12^ Malignant progression of most tumors is associated with alterations in DNA methylation, most of which occur in the early stages of the tumor.^13^, ^14^ Inhibition of oncogene promoter methylation results in loss of oncogenic function and thus promotes cancer development.^15^

Currently, most researches focused on the role of CD2AP in glomerular diseases, with little mention of the regulation of CD2AP expression in ccRCC and its prognostic value. Here, we explore the mechanisms by which CD2AP is regulated in ccRCC and its potential clinical applications. We uncovered the clinical mechanisms associated with ccRCC and molecular regulatory mechanisms, which were further confirmed by gene methylation sequencing. Moreover, we assessed the association between CD2AP and members of the immune cell population, immunomodulators and immune checkpoints in ccRCC.

## 2 RESULTS

### 2.1 CD2AP expression is downregulated in ccRCC and related to poor prognosis

**FIGURE 1:**
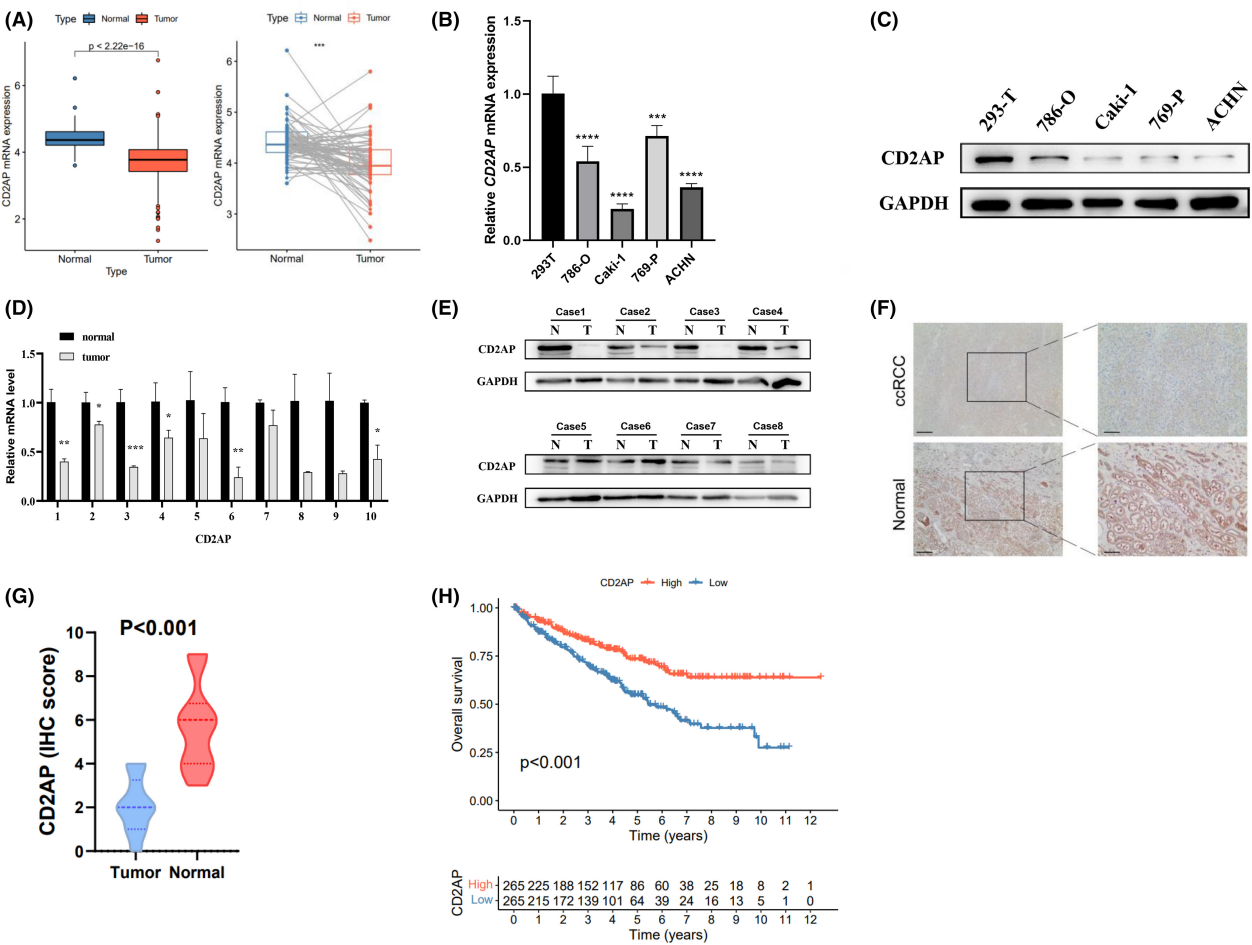
The expression pattern of CD2AP in ccRCC and adjacent normal tissues. A. relative expression levels of the CD2AP expression between ccRCC and normal tissues in TCGA dataset. B, C CD2AP mRNA (B) and protein (C) expression level in 293 T, 786‐O, 786‐P, Caki‐1 and ACHN cell lines. D–G. RT‐qPCR (D), WB (E) and immunohistochemistry (F, G) confirmed the differential expression of CD2AP in clinical tumor and paired normal tissues. H. Kaplan Meier curve comparison of overall survival rate between high and low CD2AP groups.

By analyzing ccRCC and paired normal samples from the TCGA ccRCC and GSE46699 databases, we found that CD2AP expression was markedly reduced in ccRCC compared to normal tissue (Figure 1A and Figure S1A). To validate the expression level of CD2AP, we examined this gene in different cell lines (293T, 786‐O, 786‐P, Caki‐1 and ACHN) and clinical samples, respectively. It was evident that CD2AP mRNA and protein expression were significantly down‐regulated in tumor cell lines and clinical tumor tissues (Figure 1B–G).

Subsequently, to assess CD2AP's clinical value, ccRCC samples were divided into high and low expression groups based on median CD2AP expression. Obviously, CD2AP expression correlated significantly with tumor malignancy, and its expression was significantly lower in metastatic and advanced ccRCC than in early‐stage tumors (Figure S1B). Survival analysis proved that higher CD2AP expression were associated with more optimistic patient survival, including Overall Survival (OS), Progression Free Survival (PFS) and Disease Special Survival (DSS) (Figure 1H and S1C). To verify whether CD2AP could be independent prognostic factor, we performed univariate and multivariate Cox regression analyses for OS, DSS, and PFS, respectively. In univariate Cox regression analysis model, pathological stage, grade, T, M‐staging and CD2AP expression were significantly correlated with OS, DSS and PFS (*p* < 0.001, Table 1). In a multifactorial Cox regression analysis, traditional prognostic factors, particularly grade and stage, continued to correlate well with OS, DSS, and PFS. More importantly, reduced CD2AP expression and OS, DSS and PFS were correlated significantly. Therefore, we suggested that CD2AP may be an independent prognostic factor for ccRCC and may be critical in its malignant progression.

**TABLE 1:** Univariate and multivariate cox regression analyses of CD2AP mRNA level and clinicopathological variables in predicting OS, DSS, and PFS.

Parameters		Univariate analysis				Multivariate analysis				
		HR	HR.95L	HR.95H	*p*	Coef	HR	HR.95L	HR.95H	*p*
*Overall survival (OS)*										
Age	<60	1.736	1.274	2.365	<0.001	0.464	1.591	1.165	2.173	0.004
	≥60									
Gender	male	0.941	0.687	1.288	0.705					
	female									
Stage	I&II	3.893	2.821	5.373	<0.001	1.107	3.026	1.547	5.921	0.001
	III&IV									
Grade	I&II	2.693	1.904	3.808	<0.001	0.496	1.642	1.136	2.374	0.008
	III&IV									
T	I&II	3.151	2.316	4.287	<0.001	−0.27	0.764	0.418	1.396	0.381
	III&IV									
M	M0	3.664	2.683	5.005	<0.001	0.724	2.063	1.446	2.944	<0.001
	M1&MX									
N	N0	0.883	0.653	1.194	0.417					
	N1&NX									
CD2AP		0.597	0.465	0.765	<0.001	−0.369	0.691	0.531	0.9	0.006
*Disease specific survival (DSS)*										
Age	<60	1.314	0.898	1.922	0.16					
	≥60									
Gender	male	1.227	0.811	1.856	0.333					
	female									
Stage	I&II	9.465	5.702	15.712	<0.001	1.931	6.898	3.127	15.218	<0.001
	III&IV									
Grade	I&II	4.607	2.775	7.648	<0.001	0.774	2.169	1.277	3.681	0.004
	III&IV									
T	I&II	5.303	3.491	8.055	<0.001	−0.43	0.651	0.35	1.211	0.175
	III&IV									
M	M0	7.138	4.869	10.464	<0.001	1.086	2.964	1.943	4.521	<0.001
	M1&MX									
N	N0	0.888	0.608	1.297	0.539					
	N1&NX									
CD2AP		0.461	0.345	0.615	<0.001	−0.662	0.516	0.371	0.716	<0.001
*Progression free survival (PFS)*										
Age	<60	1.227	0.896	1.681	0.202					
	≥60									
Gender	male	1.507	1.060	2.141	0.022	0.364	1.439	1.008	2.055	0.045
	female									
Stage	I&II	6.638	4.627	9.523	<0.001	1.761	5.817	3.052	11.087	<0.001
	III&IV									
Grade	I&II	3.465	2.377	5.05	<0.001	0.723	2.061	1.395	3.045	<0.001
	III&IV									
T	I&II	4.363	3.146	6.051	<0.001	−0.47	0.625	0.361	1.082	0.094
	III&IV									
M	M0	6.411	4.629	8.878	<0.001	1.165	3.207	2.237	4.598	<0.001
	M1&MX									
N	N0	0.852	0.622	1.167	0.318					
	N1&NX									
CD2AP		0.565	0.440	0.726	<0.001	−0.426	0.653	0.5	0.854	0.002

### 2.2 Promoter methylation regulated CD2AP expression and correlated with ccRCC prognosis

**FIGURE 2:**
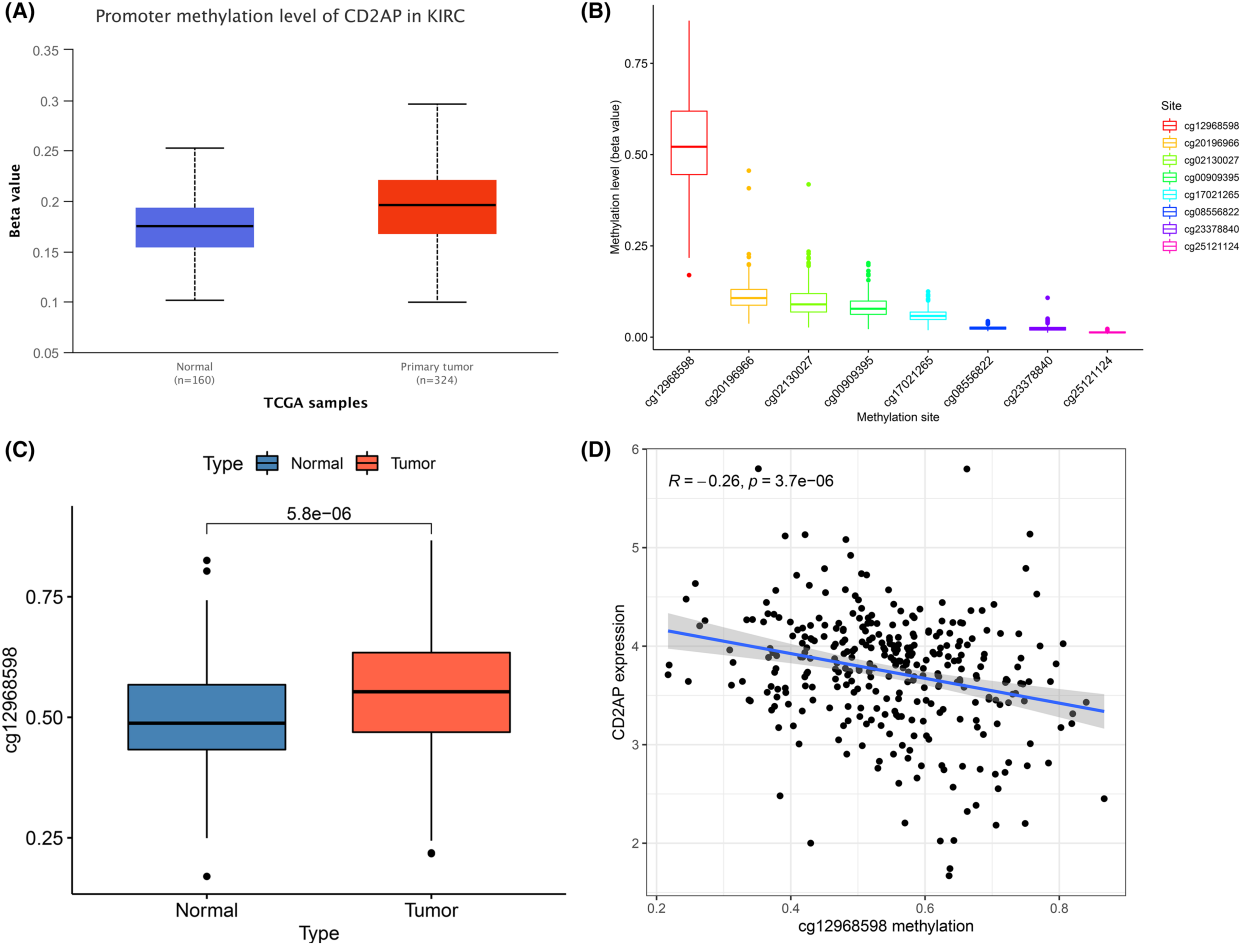
CD2AP DNA methylation status in ccRCC and adjacent normal tissues. A relative promoter methylation levels of CD2AP between the tumor and normal tissues in UALCAN dataset B Boxplot of the difference in methylation levels of 8 CpG sites in CD2AP DNA; C expression level of cg12968598 was found highly expressed in tumor compared with normal tissues; D correlation between CD2AP expression and cg12968598 methylation levels in TCGA dataset.

DNA methylation modifications as an epigenetic play a vital role in the biological engineering of gene expression regulation, DNA damage repair, and carcinogenesis.^12^, ^16^, ^17^, ^18^ Therefore, we hypothesized that hypermethylation of the CD2AP promoter may be a key factor contributing to its low expression in ccRCC. Based on UALCAN and TCGA‐ccRCC databases, we observed that CD2AP promoter methylation levels were markerly elevated in ccRCC patients compared to adjacent normal tissues (*p* < 0.001, Figure 2A). Among them, CD2AP promoter region had the highest methylation level at cg12968598 site (Figure 2B), and the methylation level of this site was significantly higher in ccRCC (Figure 2C). we found a significant negative correlation between CD2AP expression and methylation levels at this locus (*R* = −0.26, *p* < 0.001, Figure 2D). Therefore, we speculated that hypermethylation at the cg12968598 locus was probably a critical factor in the CD2AP low expression.

**FIGURE 3:**
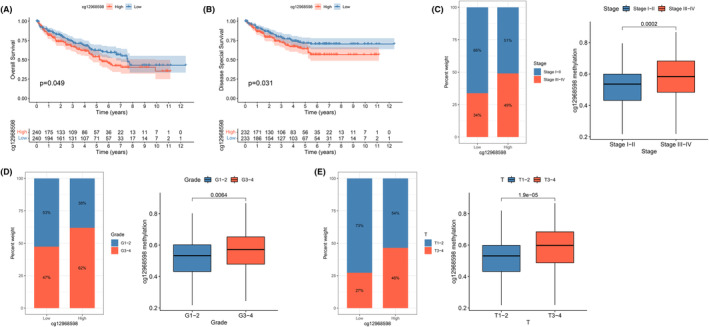
Correlation between CD2AP DNA methylation and clinical features. A, B Kaplan–Meier curves of OS (A) and DSS (B) in ccRCC patients. High methylation level groups of cg12968598 were related to shorter OS and DSS. C–E. Boxplots and the proportion for cg12968598 between different characteristics ccRCC patients, including stage (C), grade (D) and T (E).

Subsequently, we used TCGA‐ccRCC database to determine the correlation between the cg12968598 locus and ccRCC prognosis. As seen in Figure 3A,B, the prognosis (OS and DSS) of patients with cg12968598 hypermethylation was positively correlated (*p* < 0.05). Furthermore, we observed that the degree of methylation at the cg12968598 locus correlated with several clinicopathological features, including stage, grade, and T. The higher the degree of methylation at this locus, the more malignant the tumor and the greater the proportion of patients with advanced tumors; similarly, the degree of methylation at the cg12968598 locus was increased together in these more malignant ccRCCs. (Figure 3C–E). In combination with the above studies, we suggest that hypermethylation of the CD2AP promoter, particularly at the cg12968598 locus, leads to downregulation of its expression, thereby promoting malignant progression of ccRCC.

### 2.3 Validation of the hypermethylation status of the cg12968598 locus

**FIGURE 4:**
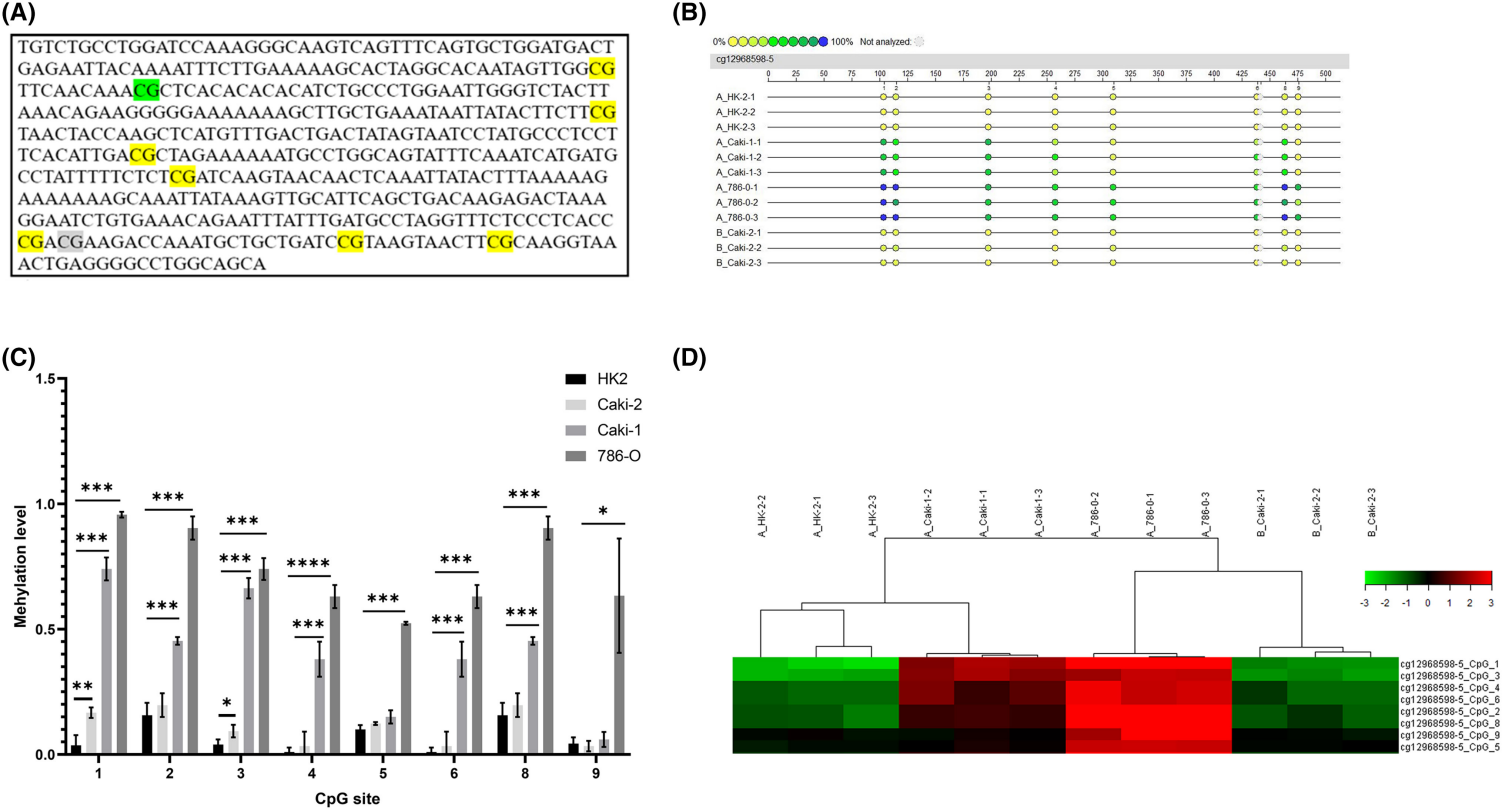
cg12968598 hypermethylation lead to low expression of CD2AP. A. CGs distribution; B. BSP results of cg12968598 methylation status in HK‐2 and ccRCC cells. C, D. Histogram (C) and percentage heat map (D) of quantitative results of methylation at cg12968598 site.

To verify the hypermethylation status of the cg12968598 site in ccRCC, we quantified the methylation level of eight CpG sites including cg12968598 by Mass ARRAY in‐flight mass spectrometry detection in HK‐2 and multiple tumor cells. Figure 4A shows the distribution of the different CpG sites. The mass spectrometry results showed that multiple CpG sites have significantly higher methylation on average in ccRCC cells than in normal kidney cells, especially in 786‐O cells (Figure 4B–D).

### 2.4 Construction of the CD2AP risk model

**FIGURE 5:**
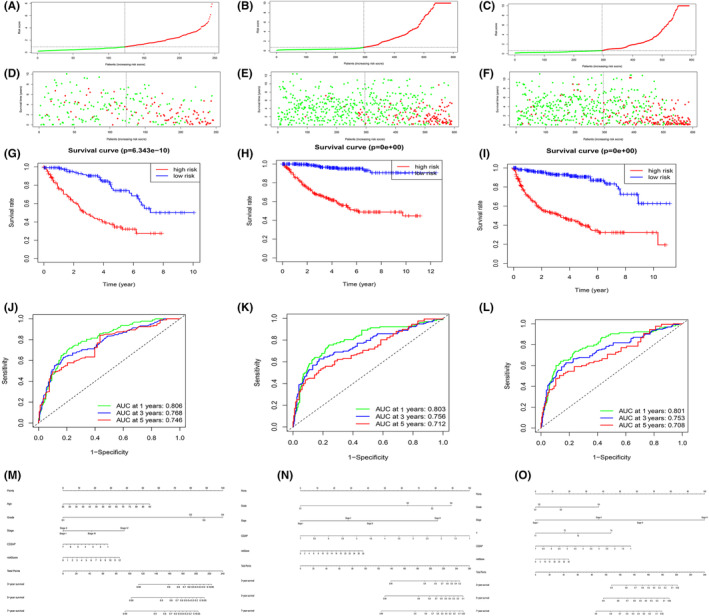
Evaluation of OS, DSS and PFS prognostic risk models in ccRCC patient based on CD2AP expression and clinical characteristics in TCGA dataset. A–C Risk score distribution of high‐risk (red) and low‐risk (green) ccRCC patients in the OS (A), DSS (B), PFS (C) model; D–F Scatter plot shows the survival status of ccRCC patients in the OS (D), DSS (E), PFS (F) model. Red dots denote patients that are dead and green dots denote patients that are alive; G–I Kaplan–Meier survival curve analysis of OS (G), DSS (H), PFS (I) in the high‐risk (red line) and low‐risk (green line) ccRCC patients;J–L Time‐dependent ROC curves show area under curve (AUC) values at 1‐, 3‐, 5‐year OS (J), DSS (K), PFS (L) in ccRCC patients; M, N nomogram to predict the OS (M), DSS (N), PFS (O) of ccRCC patients are shown.

Based on the Cox regression results, risk models were developed for OS, DSS and PFS in ccRCC, respectively. Parameters that could independently predict the prognosis of ccRCC were included in the risk model calculations (*p* < 0.05), including clinicopathological characteristics and CD2AP. We therefore calculated separate risk factors RS for different survival using the following formulae: RS for OS = (0.464 × age) + (0.396 × grade) + (1.107 × stage) + (0.496 × grade) + (0.724 × M) + (− 0.369 × CD2AP); RS for DSS = (1.931 × stage) + (0.774 × grade) + (1.086 × M) + (−0.662 × CD2AP); RS for PFS = (1.761 × stage) + (0.723 × grade) + (1.165 × M) + (−0.426 × CD2AP). Patients were divided into low and high groups based on median scores. First, we ranked the patients' OS, DSS, and PFS risk scores and analyzed the distribution (Figure 5A–C). Dot plots displayed the OS, DSS, and PFS status (Figure 5D–F). These figures revealed that the higher the risk score, the more patients in high subgroup and the more deaths. To further validate the predictive properties of RS in ccRCC for OS, DSS, and PFS, K‐M curves were constructed to compare the different survival times between two groups. Low‐risk patients had a higher OS (*p* < 0.001, Figure 5G–I). We then used time‐dependent ROC curves and column plots to visualize the predictive performance of 3 prognostic models (Figures 5J–O). Area under the curve (AUC) >0.7 indicated that RS is effective in predicting prognosis.

### 2.5 Correlation of CD2AP expression with immune infiltration

Upon ascertaining the prognostic value and clinical relevance of CD2AP in ccRCC, we performed an analysis of the correlation between CD2AP and immune cell infiltration using TIMER database. Copy number alteration (CNA) type of CD2AP correlated significantly with several immune cell infiltrations (Figure S2A). Moreover, we observed a positive correlation between CD2AP expression and infiltration of several immune cells, particularly macrophages and neutrophils (cor >0.3, *p* < 0.001, Figure S2B). Of these, CD2AP expression was most strongly correlated with macrophages, suggesting that CD2AP may regulate the ccRCC immune microenvironment mainly through macrophages. However, the survival analysis of infiltrating immune cells with CD2AP expression was not statistically significant (Figure S2C).

Subsequently, we analyzed the correlation between CD2AP expression and lymphocytes, immunomodulators and chemokines through TISIDB database. Figure S3A displayed the correlation between CD2AP and tumors infiltrating lymphocytes. Figures S3B–G displayed the correlation between CD2AP expression levels and immunomodulatory agents including immunosuppressants, immunostimulants and major histocompatibility complex molecules. Among these, we listed the more common immunosuppressive agents currently available, including PDCD1 (*ρ* = − 0.267, *p* < 0.001), CD274 (*ρ* = 0.293, *p* < 0.001), CDLA4 (*ρ* = −0.22, *p* < 0.001) and LAG3 (*ρ* = −0.291, *p* < 0.001) (Figure S3C). These results indicated that CD2AP was likely to be participating in the co‐regulation of the tumor microenvironment by these immune molecules.

## 3 DISCUSSION

CD2AP is mainly located in the podocyte slit membrane in kidney and is able to maintain podocyte physiological function.^9^ When CD2AP expression is reduced or gene mutations occur, glomerular podocyte function is impaired, with massive proteinuria and aggravating renal disease.^19^ Currently, there are fewer studies on whether CD2AP is associated with ccRCC diagnosis and prognosis. Therefore, this study was mainly to determine whether CD2AP expression could suggest the prognosis of ccRCC patients.

In this work, based on TCGA‐ccRCC database and clinical samples, we found that CD2AP expression was significantly lower in ccRCC and that low CD2AP expression was positively correlated with the malignancy of ccRCC, with reduced expression predicting poorer survival in ccRCC patients. Multivariate COX regressions also suggest that CD2AP may be an independent risk factor for ccRCC prognosis. To investigate the cause of reduced CD2AP expression in ccRCC, after processing TCGA‐ccRCC methylation data, we found that the CD2AP promoter was most highly methylated at the cg12968598 site and confirmed that this site was the main cause of low CD2AP expression by methylation mass spectrometry sequencing.

Currently, it has been demonstrated that CD2AP can be used as a marker for some immune cells, such as plasmacytoid dendritic cells.^20^ Similarly, by mining the TIMER database, we found that CD2AP is associated with immune cell infiltration, especially macrophages, which is important for the formation of the tumor immune microenvironment. Moreover, most studies have found that large numbers of immune cells induce the formation of ccRCC immune microenvironment, which affects patient survival and prognosis.^21^, ^22^ Clinically, metastatic ccRCC patients can be treated with immune checkpoint inhibitors, such as anti‐PD‐1, CTLA‐4 drugs.^2^, ^23^, ^24^ Using TISIDB database, we also found a statistical correlation between CD2AP and immunosuppressive molecules. These results suggest that CD2AP can modulate the extent of tumor immune infiltration by affecting immune cells and immune molecules. Therefore, we believed that CD2AP could be used as a clinical target to modulate tumor immune microenvironment, and could be applied to combine with immune checkpoint inhibitors to improve ccRCC prognosis. Furthermore, CD2AP was originally cloned in T cells, and the expression level of CD2AP can regulate T cell signal transduction.^6^, ^25^ Therefore, the role of CD2AP in tumor immune microenvironment needs to be further explored.

Our study has limitations to some extent. This work was mainly a retrospective study based on public databases and was partially biased. Relationship between CD2AP and immune cells and molecules was derived from public databases, which are limited in terms of the types of immune cells and immune molecules and lack further validated clinical data. Future, we will continue to explore the potential molecular mechanisms of CD2AP in the context of immunotherapy, aiming to provide new directions and strategies for targeted molecular therapy in ccRCC patients.

## 4 CONCLUSION

Our results indicated that CD2AP can be an effective prognostic factor for ccRCC. Low CD2AP expression was significantly correlated with the development of ccRCC and poor prognosis. CD2AP promoter methylation was the main cause of its low expression. This study provides new therapeutic targets and ideas for the clinical treatment of ccRCC.

## 5 MATERIALS AND METHODS

### 5.1 Sample data acquisition

We used UCSC Xena browser (https://xenabrowser.net/) to obtain CD2AP expression RNA‐seq data, clinicopathological data, patient survival information and DNA methylation data for all samples in the TCGA‐ccRCC database.^26^, ^27^ Considering overall survival (OS), disease‐specific survival (DSS) and progression‐free survival (PFS) as the primary outcomes for ccRCC patients, we further screened OS data for 531 primary ccRCC patients, DSS data for 520 ccRCC patients, and PFS data for 529 patients.

### 5.2 Cell culture

Human renal epithelial cells 293T, and several ccRCC cell lines (786‐O, 786‐P, Caki‐1 and ACHN) were used for this work. 293T cells and ACHN were cultured in DMEM, 786‐O and 786‐P cells in 1640, Caki‐1 cells in McCoy's 5A medium was used for culture. All media were supplemented with 10% fetal bovine serum and 1% penicillin/streptomycin reagent. The cell lines were cultured at 37°C in a humidified incubator with 5% CO2.

### 5.3 Real‐time quantitative PCR

Total RNA was extracted from the cell lines using Trizol reagent. cDNA was reversed to cDNA using a reverse transcription kit. cDNA was synthesized for qRTPCR analysis. Primer specificity was tested by melting curve analysis and gel electrophoresis. Expression values were normalized to the endogenous control GAPDH and amplification efficiency using the adjusted 2^−ΔΔCt^ method. Primer sequences are shown in Table S1.

### 5.4 Western Blot

Protein extracts were prepared using 1 × RIPA buffer with a protease/phosphatase inhibitor mixture. SDS‐PAGE was performed on total protein and transferred to PVDF membranes. After closure with 5% BSA in Tris‐buffered saline‐Tween‐20, the membranes were incubated with CD2AP (Cell Signaling Technology, 5478S) and GAPDH (abcam, ab9485) primary antibodies at 4°C overnight, dilution‐labeled HRP secondary antibody was added and incubated for 1 h at room temperature and DAB developed.

### 5.5 Immunohistochemistry

Paraffin samples from clinical 10 pairs of ccRCC and paraneoplastic tissue were applied for immunohistochemistry. This study was approved by the Ethics Committee of the First Affiliated Hospital of Nanjing Medical University (No.2022‐SR‐708). The slides were dewaxed and hydrated prior to staining. Antigens were recovered by boiling the sections in Tris‐EDTA buffer (pH 9.0). Sections were incubated at 4°C with CD2AP primary antibody and horseradish peroxidase‐coupled secondary antibody. After incubation, the sections were washed three times in PBS buffer, dried, and then retouched with a drop of DAB reagent for color development and hematoxylin. The scoring system was based on the intensity and degree of staining: intensity of staining was classified as 0 (negative), 1 (weak), 2 (moderate), and 3 (strong); the degree of staining depended on the percentage of positive cells (examined in 200 cells) and was classified as 0 (<5%), 1 (5%–25%), 2 (26%–50%), 3 (51%–75%) and 4 (>75%). The degree of staining as well as the percentage of positivity were scored separately and multiplied together to obtain a composite score.

### 5.6 DNA methylation analysis

The Agena MassARRAY® platform (CapitalBio technology, Beijing) is used to detect methylation status of the promoter CpG site of CD2AP in multiple cells. The assay involved bisulfite conversion, PCR amplification, in vitro transcription and RNase A‐specific digestion and matrix‐assisted laser desorption ionization time‐of‐flight mass spectrometry. The experimental procedure converts the DNA template CpG methylation state into sequence differences in the RNA enzyme section, which are distinguished by differences in molecular weight as determined by mass spectrometry. A special analysis software, MassArray EpiTYPER, was applied to give the CpG methylation status of the DNA fragments. Primers were designed using Sequenom's Epidesigner (http://www.epidesigner.com) (Table S1). This study mainly contained a total of eight valid CpG loci.

### 5.7 Immune‐related analysis

The TIMER database (https://cistrome.shinyapps.io/timer/) analyses RNA‐Seq expression profiling data of tumor samples in TCGA, and correlate tumor‐infiltrating immune cells with expression, mutations, and somatic copy number variation.^28^ TISIDB database (http://cis.hku.hk/TISIDB/) documents nearly 1000 genes associated with anti‐tumor immunity, T‐cell killing and immunotherapy, and also calculates the correlation of genes with immune molecules.^29^

### 5.8 The establishment of the risk model

Using multivariate COX regression analysis, we screened for independent prognostic factors and developed prognostic models for OS, DSS and PFS based on these variables, calculating risk scores (RS) based on the *α*1 × clinical factor 1 + *α*2 × clinical factor 2 + … + *α*n × CD2AP equation. Where *α* is the correlation coefficient between clinical factors and CD2AP.

### 5.9 Statistical analysis

All statistical analyses were done using R software (version 4.0.3). “survivor” and “survminer” were applied to assess survival differences. Chi‐square test was used to assess the correlation between CD2AP and numerous clinical factors. Kaplan–Meier curves and log‐rank tests were used to compare the effect of CD2AP expression and RS on patient survival.^30^ ‘timeROC’ package is used to plot subject operating characteristic curves (ROCs) and assess the accuracy of CD2AP and RS in predicting ccRCC prognosis. The “rms” package is used to plot prognostic line graphs. *p*‐values <0.05 are considered statistically significant.

## AUTHOR CONTRIBUTIONS

**Can Chen:** Conceptualization (equal); formal analysis (equal); investigation (equal); methodology (equal); software (equal); writing – original draft (equal). **Jia Xu:** Conceptualization (equal); formal analysis (equal); investigation (equal); methodology (equal); resources (equal). **Jie‐Xin Zhang:** Conceptualization (equal); data curation (equal); formal analysis (equal); investigation (equal); methodology (equal); project administration (equal). **Lin‐Yuan Chen:** Conceptualization (equal); formal analysis (equal); methodology (equal); resources (equal); validation (equal). **Yu‐Ang Wei:** Data curation (equal); formal analysis (equal); investigation (equal); resources (equal). **Wei‐Ming Zhang:** Conceptualization (equal); data curation (equal); methodology (equal); resources (equal); supervision (equal). **Peng‐Fei Shao:** Conceptualization (equal); data curation (equal); formal analysis (equal); methodology (equal); resources (equal); supervision (equal). **Hua‐Guo Xu:** Conceptualization (lead); formal analysis (lead); funding acquisition (lead); supervision (lead); writing – review and editing (lead).

## FUNDING INFORMATION

This study was supported by Natural Science Foundation of Jiangsu Province of China (BK20181492), the National Key Clinical Department of Laboratory Medicine of China in Nanjing, Key laboratory for Laboratory Medicine of Jiangsu Province (ZDXK202239) and by the Priority Academic Program Development of Jiangsu Higher Education Institutions.

## CONFLICT OF INTEREST STATEMENT

None.

## ETHICS STATEMENT

The human ethics involved in this study was approved by the Ethics Committee of the First Affiliated Hospital of Nanjing Medical University. The patient provided written informed consent to participate in this study.

## CONSENT FOR PUBLICATION

Not applicable.

## Supporting information


Appendix S1.

## Data Availability

The data that support the findings of this study are available from the corresponding author upon reasonable request.
