# Supplementary material for: CD2AP is a potential prognostic biomarker of renal clear cell carcinoma
Source: Cancer Med. 2024 Mar 8;13(4):e7055. doi: 10.1002/cam4.7055 (PMC10923042; doi:10.1002/cam4.7055)
Supplement: Supplementary file 1 — Appendix S1. [file CAM4-13-e7055-s001.docx]

Supplementary Appendix

This appendix has been provided by the authors to give readers additional information about their work. Supplement to: CD2AP Is a Potential Prognostic Biomarker of Renal Clear Cell Carcinoma

**CD2AP Is a Potential Prognostic Biomarker of Renal Clear Cell Carcinoma carcinoma**

***Supplemental Materials***

**Table of Contents**

**Table S1** The sequences of primers.

**Figure S1** The expression of CD2AP was down regulated in ccRCC and related to various clinicopathological features.

**Figure S2** CD2AP is closely related to immune infiltration in ccRCC (TIMER).

**Figure S3** Spearman’s correlation of CD2AP with lymphocytes, immunomodulators and chemokine (TISIDB).

| **Table S1** **The sequences of primers.**   \|  \| **Sequence Description** \| **Sequence** \| \| --- \| --- \| --- \| \| qPCR \| CD2AP-F \| TGGAGATAACAAAAACAGATACCGA \| \| CD2AP-R \| TTCAGGCTTTGGAGCTGGAG \| \| GAPDH-F \| CGGAGTCAACGGATTTGGTCGTAT \| \| GAPDH-R \| AGCCTTCTCCATGGTGGTGAAGAC \| \| BSP \| cg12968598-5-F \| aggaagagagTGTTTGTTTGGATTTAAAGGGTAAG \| \| cg12968598-5-R \| cagtaatacgactcactatagggagaaggctTACTACCAAACCCCTCAATTTACCT \| |
| --- | --- | --- | --- | --- | --- | --- | --- | --- | --- | --- | --- | --- | --- | --- | --- | --- | --- |


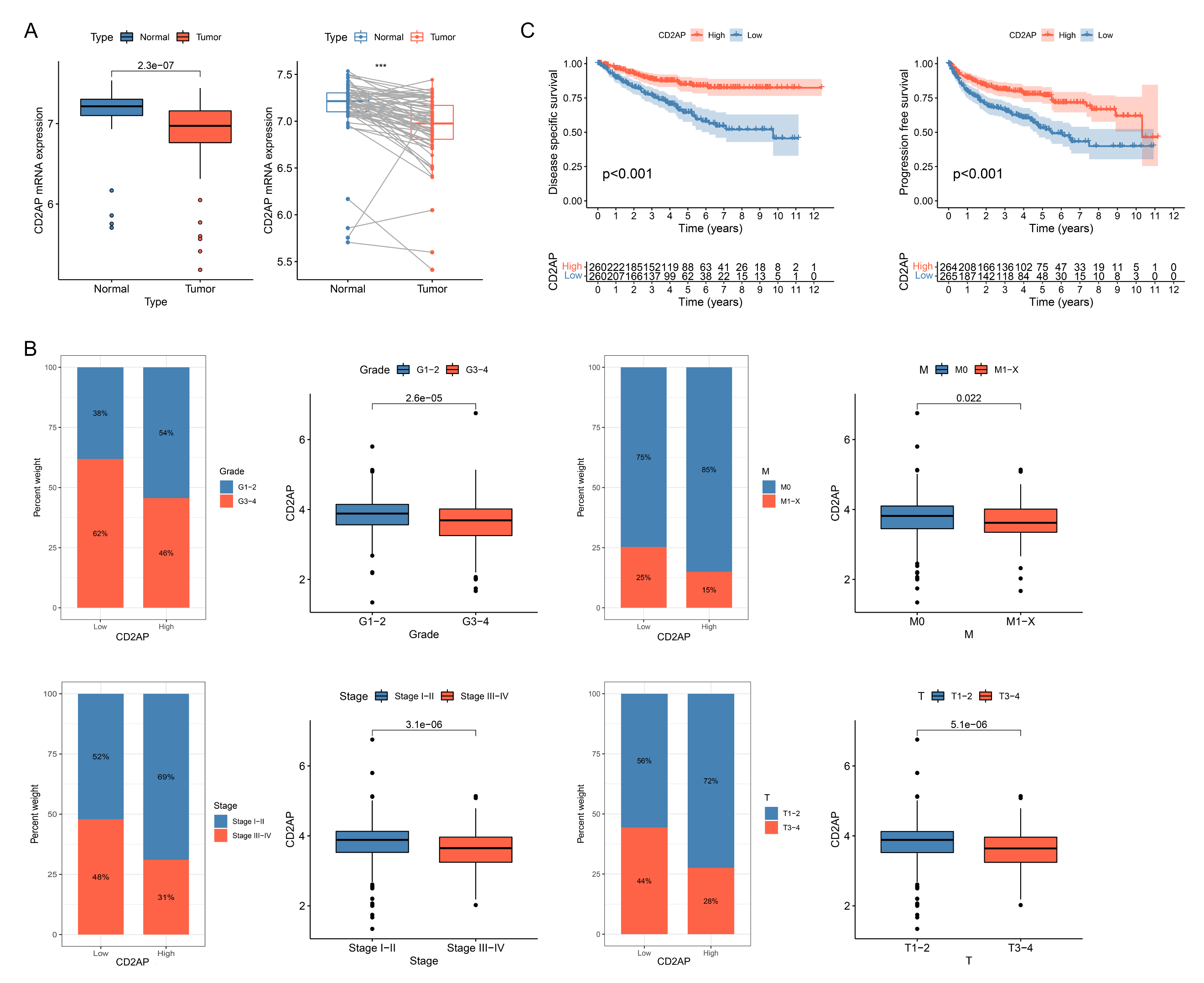


**Figure S1 The expression of CD2AP was down regulated in ccRCC and related to various clinicopathological features. A.** relative expression levels of the CD2AP expression between ccRCC and normal tissues in GSE46699 dataset; **B.** Boxplots and the proportion for CD2AP between different characteristics ccRCC patients, including stage, grade, T and M; **C.** Kaplan-Meier survival curves comparison of high and low expression of CD2AP for ccRCC patients. Disease-specific survival on the left and progression free survival on the right.


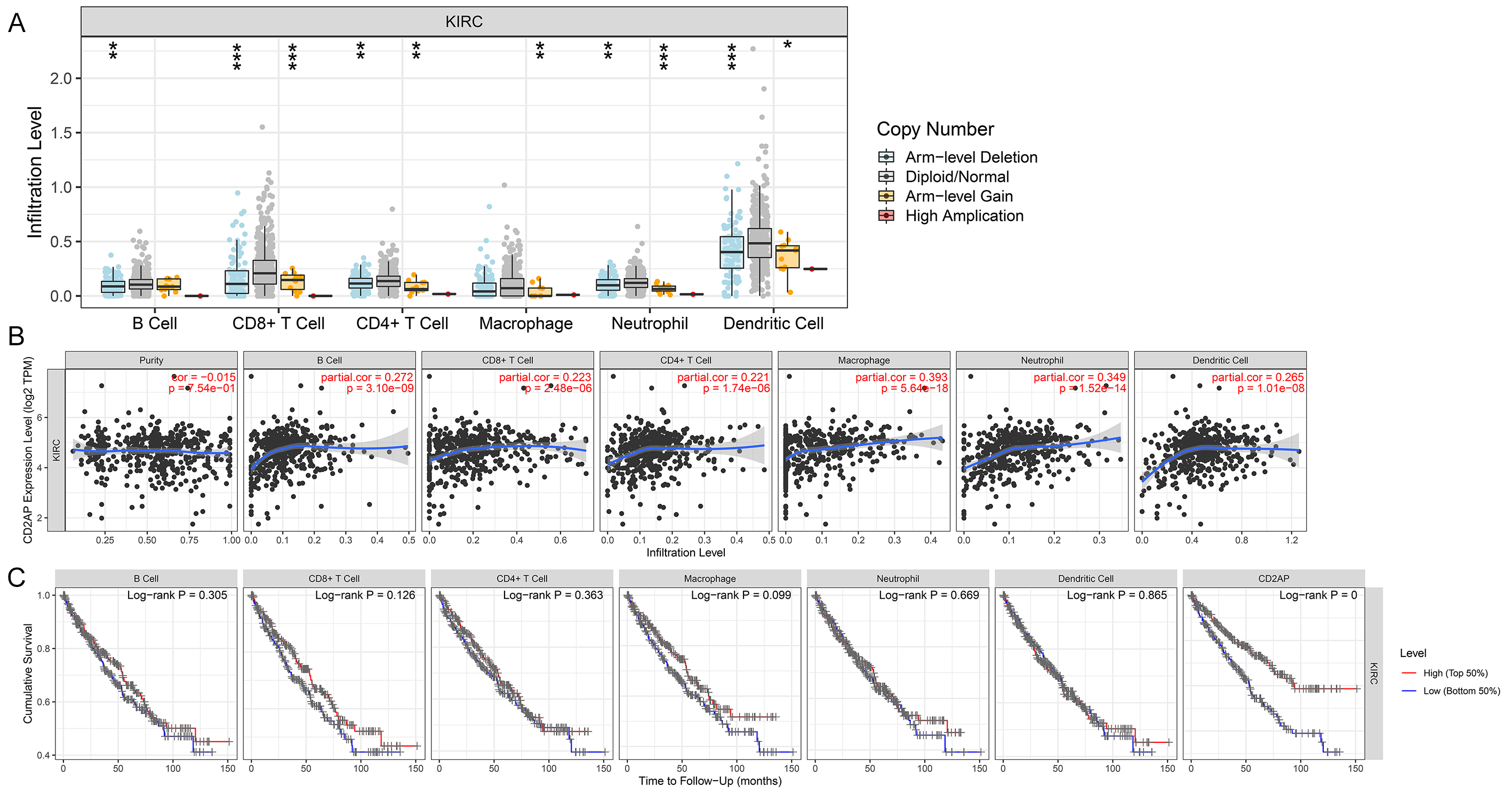


**Figure S2** **CD2AP is closely related to immune infiltration in ccRCC (TIMER). A.** CNA of CD2AP was significantly correlated with immune infiltration levels of several immune cell types in ccRCC; **B.** correlation of CD2AP expression with immune infiltration level of immune cells in ccRCC; **c** Kaplan-Meier survival analysis of immune cells. CNA: copy number alterations; *P<0.05, *P<0.01, *P<0.001


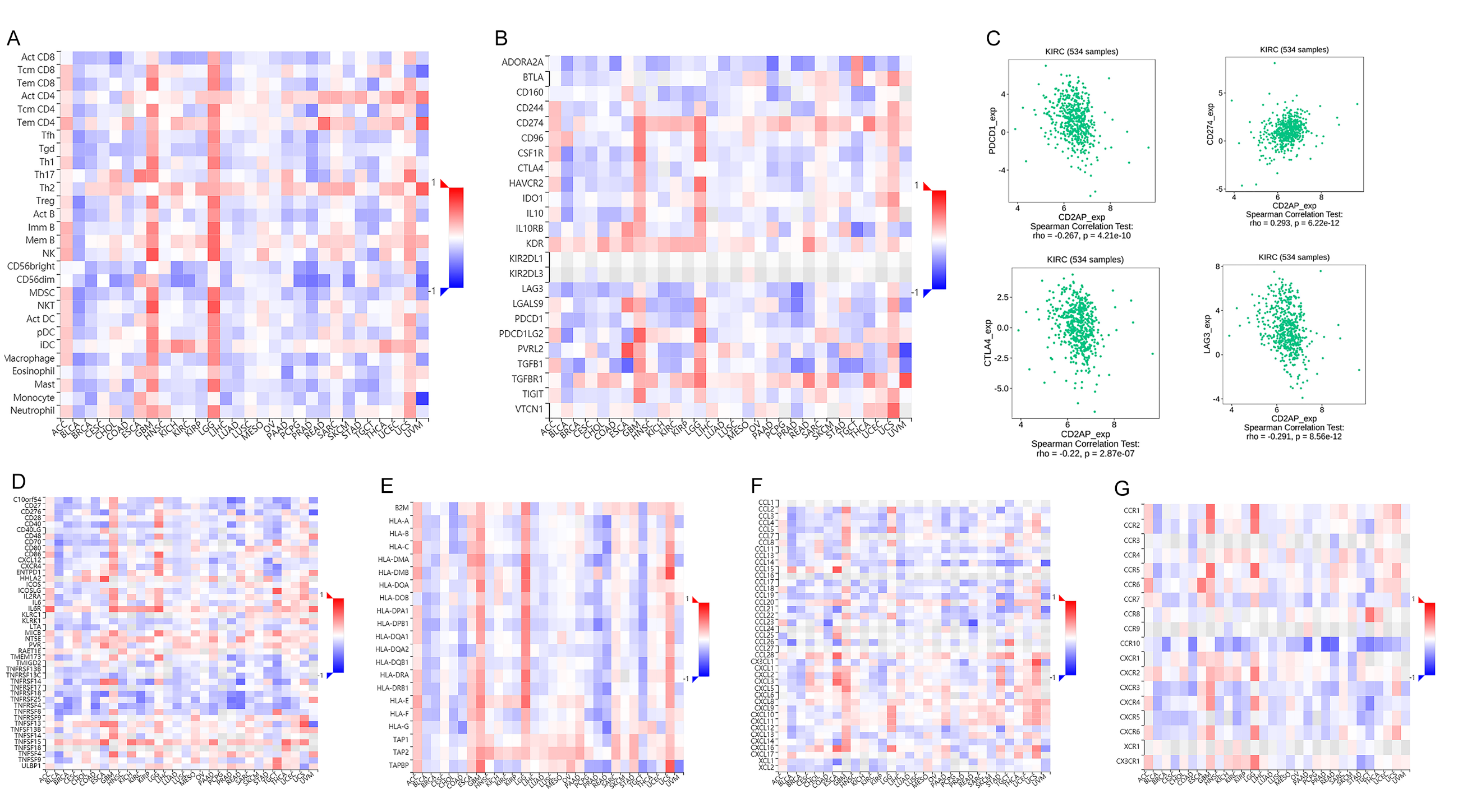


**Figure S3** **Spearman’s correlation of CD2AP with lymphocytes, immunomodulators and chemokine (TISIDB).** **a** Relations between the abundance of TILs and CD2AP expression; **b** Relations between the abundances of immunoinhibitors and CD2AP expression; **c** Representative immunoinhibitors display a strong Spearman’s correlation with CD2AP expression; **d** Relations between abundances of immunostimulators and CD2AP expression; **e** Relations between abundance of MHC molecules and CD2AP expression. **f** Relations between abundance of chemokines and CD2AP expression; **g** Relations between abundance of receptors and CD2AP expression. Red and blue cells indicate positive and negative correlations, respectively. The color intensity is directly proportional to the strength of the correlations. TILs: tumor-infiltrating lymphocytes; MHC: major histocompatibility complex
